# 

**Published:** 1860-07

**Authors:** 


### CONTENTS.

ORIGINAL ESSAYS AND COMMUNICATIONS.

New Fusible Alloys. By B. Wood, M. D. .... 1
Dental Surgery. By P. G. C. Hunt .... 5
Gutta Percha for Separating and Regulating Teeth. By B. Wood .... 10
A New Method of using Vulcanite. By P. G. C. Hunt .... 12
Language—Mistakes—Corrections .... 16
Vulcanite Ideas .... 18

PROCEEDINGS OF SOCIETIES.

Discussions of the Kentucky State Dental Association .... 19

Correspondence .... 32

Selections .... 36

Associations .... 55

Editorial .... 69

